# A new approach for serial electron diffraction data collection

**DOI:** 10.1107/S2052252523010953

**Published:** 2024-01-01

**Authors:** Brent L. Nannenga

**Affiliations:** a Chemical Engineering, School for Engineering of Matter, Transport and Energy, Arizona State University, Tempe, AZ 85287, USA; b Center for Applied Structural Discovery, The Biodesign Institute, Arizona State University, Tempe, AZ USA

**Keywords:** serial electron diffraction, scanning transmission electron microscopy, structure determination, nanocrystallography, beam-sensitive materials, zeolites

## Abstract

This commentary describes a novel method for serial electron diffraction data collection in electron crystallography, utilizing a scanning transmission electron microscope to rapidly obtain patterns with low radiation dose. This approach, demonstrated with zeolite samples, has the potential to provide highly automated and rapid structures from nanocrystalline materials.

From materials characterization to the study of biological materials, electron crystallography has played a key role in the structural sciences (Zou *et al.*, 2011 ▸; Glaeser, 2007 ▸). The wider field of electron crystallography has always been driven forward by advances in methodology. In recent years, electron crystallographic methods that make use of three-dimensional micro- and nanocrystals have come to the forefront (Nannenga & Gonen, 2019 ▸; Gemmi *et al.*, 2019 ▸). Currently, the most common method of data collection is continuous rotation of the crystal in the electron beam, which produces electron diffraction datasets analogous to single-crystal X-ray diffraction datasets (Nannenga *et al.*, 2014 ▸; Cichocka *et al.*, 2018 ▸; Gemmi *et al.*, 2015 ▸). This robust approach for continuous rotation electron diffraction data collection has led to an explosion in popularity of the technique and it has been successfully applied to a variety of samples including nanomaterials, organic molecules, pharmaceuticals, peptides and proteins.

Despite growing interest in the field, there are portions of the data collection pipeline that are the focus of continued methods development. For example, when studying samples that are beam sensitive and require large amounts of data to be collected, structure solution can prove to be difficult. Therefore, improvements to data collection throughput, automation of the data collection process and minimizing the exposure of crystals during collection of diffraction datasets are areas of interest.

In this issue of **IUCrJ** , Hogan-Lamarre *et al.* (2024 ▸) directly address these issues by utilizing scanning transmission electron microscopy (STEM) to rapidly collect serial electron diffraction patterns with low dose to determine high-resolution structures of nanomaterials. This work builds on previous serial electron diffraction methodology that made use of a customized STEM setup and hybrid pixel detector to determine the structures of model proteins from nanocrystals (Bücker *et al.*, 2020 ▸). A major benefit of this new method (Fig. 1 ▸) is that, in contrast to the previous study, it does not require specialized STEM hardware, makes use of the microscope scripting software provided and can be performed using more widely available electron microscope camera systems. These improvements will make the implementation of this technique more broadly accessible to the electron crystallography community. The STEM serial electron diffraction workflow starts by screening the grid at low magnification with minimal dose delivered to the sample. Using the low-magnification screening, regions of interest (*i.e.* potential crystals) are identified and selected. Each region of interest is then targeted, and a single electron diffraction pattern is collected. The data are then processed and combined to produce serial electron diffraction datasets for subsequent data processing and structure determination efforts.

As a proof of concept for this new serial electron diffraction workflow, the authors of the study used two zeolite samples. The first sample, zeolite Y, was used to benchmark the new serial diffraction methodology as it could be directly compared with data collected by standard continuous rotation data collection. With both approaches, a high-resolution structure of zeolite Y was determined with similar quality, thereby validating the serial diffraction approach on this model target. The next target chosen for validation was the zeolite ZSM-25, which represented an exciting sample for the new methodology as electron diffraction had not yet been able to produce datasets of sufficient quality for *ab initio* structure solution. With the new approach in hand, the authors were able to collect serial electron diffraction data from ZSM-25 and ultimately generate a 99.8% complete dataset to 0.90 Å, which facilitated structure solution by direct methods. This demonstration of the first ever *ab initio* structure solution of ZSM-25 highlights the power of STEM serial electron diffraction data collection, and the more user-friendly implementation promises to allow more users to take advantage of the technique.

Serial electron diffraction data collection using STEM is an important new approach for the field of electron crystallography. It will be exciting to follow future work in this area including the continued streamlining of the STEM workflow, the extension of the technique to an even broader range of STEM and detector combinations, and the structure determination of more novel targets using the methodology. The possibilities of further combining STEM serial electron diffraction with existing continuous rotation methods will also be interesting to developers and users. Overall, STEM serial electron diffraction has the potential to provide highly automated and rapid structures from nanocrystalline materials that may have proven difficult to study using other structural methods.

## Figures and Tables

**Figure 1:**
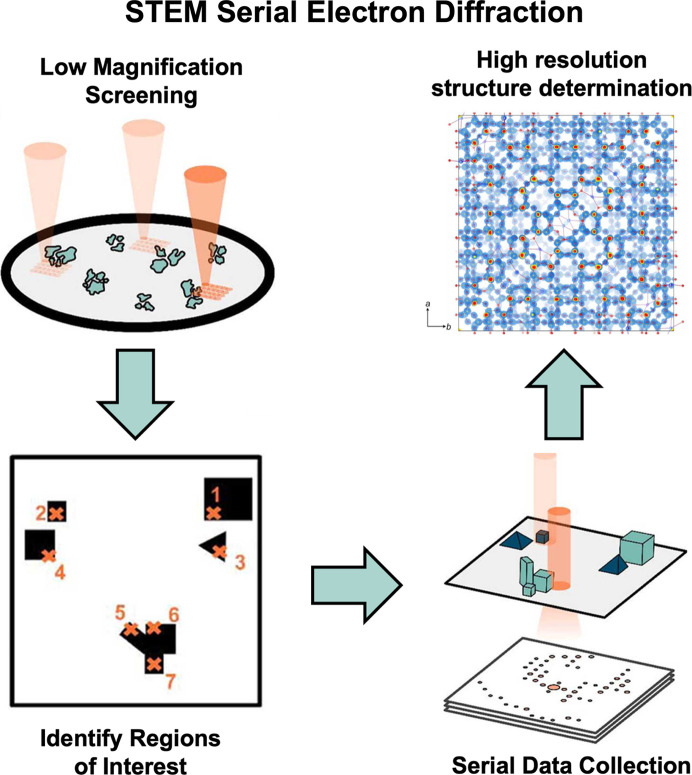
Overview of the STEM serial electron diffraction workflow presented by (and adapted from) Hogan-Lamarre *et al.*, (2024 ▸).
